# Revisiting frameworks: Have they helped us reduce disaster risk?

**DOI:** 10.4102/jamba.v15i1.1491

**Published:** 2023-12-30

**Authors:** Ben Wisner, Irasema Alcántara-Ayala

**Affiliations:** 1 Institute for Risk and Disaster Reduction, University College London, England, United Kingdom; 2 Environmental Studies Program, Oberlin College, Oberlin, OH, United States; 3 Institute of Geography, National Autonomous University of Mexico (UNAM), Mexico City, Mexico

**Keywords:** disaster risk frameworks, policymaking and practice, disaster risk management, disaster risk reduction, responsibility, accountability, effective disaster risk governance

## Abstract

Owing to the increasing impact of disasters worldwide, there is a recognised need for putting theory into relevant and effective practice in disaster risk reduction (DRR). Substantial research has been oriented on reducing loss of life, injury, livelihoods and infrastructure disruption. In this paper, we gathered the main insights derived from the reflections of the earlier work of authors or co-authors of frameworks for understanding disaster risk.

**Contribution:**

The findings add to our understanding of how bridging the gap between science and policymaking to manage disaster risk is shaped. This review showed evidence that disaster risk research and interaction among relevant DRR stakeholders have evolved. The degree of decisions, resource allocations and actions of state and non-state actors are influenced by applying such frameworks. However, limitations on resources in the policy-making spheres may require prioritisation but also new challenges in terms of responsibility, accountability and effective disaster risk governance.

## Introduction

The contributions we have solicited from friends and colleagues are reflections on their earlier work as authors or co-authors of frameworks for understanding disaster risk. The earliest one dates from 2001. We asked contributors to consider the reception and application of the framework as well as to comment with the benefit of hindsight on criticisms this work has received and gaps and opportunities left unaddressed.

The question we pose as the subtitle of our editors’ introduction is central and critical: have these frameworks helped to reduce loss of life, injury, livelihoods and disruption of infrastructure? In more general terms: how does one get from theory to relevant and effective practice? So, if practice at a local scale erases academically or bureaucratically imagined boundaries between theory and practice, what is the use of ‘theory’ at all? To answer this question, we have to consider what theories, models and frameworks do. There are those that are normative and provide direction to practitioners on the basis of *a priori* propositions derived from the core axioms of a body of knowledge such as economics or psychology. Thus, neoliberal economics might insist that the economic benefit of risk reduction practice must be greater than its cost. Psychology may insist that an individual’s adoption of a risk reduction practice is based on the satisfaction of a basic need and must not conflict with the satisfaction of other basic needs. By contrast, theory, models and frameworks may also be the result of empirical research on risk reduction decision-making and adoption of practices. This is an *a posteriori* approach that results in the generalisation that in turn can be used normatively in designing and employing practices. One might think of the *a priori* approach as resulting in theory-as-axiom, while the *a posteriori* approach as defining theory-as-generalisation.

We and our theme issue contributors do not hold to either of these approaches. For our purposes, frameworks are nothing more or less than checklists of the kind a pilot uses to make sure all system function before flight or the mental checklist used by a physician in the differential diagnosis in order to make sure that even improbable aetiologies are considered. While this sounds a lot like the theory-as-axiom approach, the essential difference lies in *lay people’s active participation* in testing each axiom-based response against their own experience. The result is a dynamic balance of *a priori* and *a posteriori* approaches.

Participation can be a weary buzzword. So, we have to be clear about what we mean. One can think of a ladder of participation. The first, lowest rung is a simple *pronouncement* to local people of decisions already made by the government. ‘A cyclone shelter will be built’. ‘Everyone will contribute labor and/or cash to build it’. Higher on the ladder is *consultation*. ‘Government has decided you need a cyclone shelter here. Let us discuss how it can be funded and built’. These are both kinds of *instrumental participation*. The problem has been externally pre-selected by authorities and experts. Still higher on the ladder is *transformative* participation (Wisner 1988). In this case, the outside expert brainstorms with local people who are respected as lay experts or inside experts. Cyclone history (especially oral history) in the locality and its impacts are put in the context of all the other hazards faced locally and local aspirations and resources are reviewed. It is not a foregone conclusion that the first priority will be to build a cyclone shelter.

Taking the transformative view of participation, the assemblage of items in the pre-flight checklist provides the framework for understanding and reducing the risk that must be generated in a dialogue between the outside and inside experts. What goes on at the local scale (what we call practice) is dependent on vigorous local dialogue and co-production of risk assessment and interventions. Despite the tendency for upscale institutions to divide mandates and budgets by ‘sectors’, at the hyperlocal scale, practice inevitably cuts across sectors.

## The labyrinth of definitions

One of the most basic ways that top-down power is exerted in the domain of disaster policy and practice is an insistence on received definitions. Ian Davis has this to say about definitions of disaster in his comprehensive review of progress in DRR (Davis & Alexander 2022:18–19):

There are now more than 50 easily accessible glossaries of disaster terminology. A by-product of having a vigorous debate about definitions is the plethora of definitions that result from examining every angle under which a phenomenon can be viewed.

There are four possible solutions to this unhappy state of affairs, but they all have drawbacks (Kelman 2013):

- Do without definitions. The result of following this strategy is … misestimation, misapprehension and failure to communicate …
- Impose definitions. Unfortunately, people are bound to be dissatisfied with the result of this approach as it restricts the freedom of choice.
- Use working definitions. For the sake of achieving a temporary consensus, debate is suspended and commonly agreed definitions are used …
- Use more complex approaches, with negotiation and mediation. This is a time-consuming strategy that will only work if an agreement is reached.

The ‘complex approach’ involving negotiation and mediation is precisely what transformational participation should achieve. However, there are many diverse actors involved in a disaster situation, and each brings with them a ‘culture’ that may or may not be compatible with the last option. (Revet & Langumier 2015). Immediate responders may intervene without a definition that provides appropriate scope and scale and end up inundating the affected people and local governments. In the words of the World Health Organization and Pan American Health Organization (2022):

Receiving the donations that pour into a disaster-stricken country is not an easy task. The flow of solicited and unsolicited relief supplies is often so overwhelming that it exceeds the ability of the country or an NGO to handle it.

## What does it mean to ‘frame’?

All thinking and writing make assumptions about reality. Most of these are unconscious and tacit. Some are made consciously and with intention. We are more concerned with the latter type. The word implies delimitation or the establishment of boundaries and limits. It is about context. Events, things and people are grouped or corralled in the mind; they are thrown into juxtaposition. The metaphors that come to mind are spatial. The person who frames is choosing a space in which an aspect of reality – say disasters – is thought about and discussed. To choose or to offer one space is to deny another. An obvious example in the case of disaster discourse is to deny a linguistic space in which disasters are ‘natural’, in which there is an identity between hazard and disaster. This framing would deny linguistic space to the proposition that a flood (a hazard event in nature) is a disaster for everyone, not just for those living close to the river or those without insurance. Yet, interestingly, it may be the case that all frameworks have trap doors or hidden exits at least available to the imagination. In a society populated by Tronto’s caring individuals (1993), one can imagine that the flood is experienced as everyone’s disaster.

## What does it mean to ‘reflect’?

We have invited authors to think again, to revisit themselves when they thought and wrote what they did, to see themselves in a mirror that throws back a self that once thought and wrote. Is that self still recognisable in the mirror? Looking back at some things one of us wrote in the 1970s and 1980s, we exclaim, ‘how could I have thought that?’. We might like to ‘turn back the clock’ and un-do mistakes and forestall misapplications of the kind Malm (in this volume) identifies in the youth, adolescence and maturity of the ‘progression of vulnerability’ (PAR) framework (Wisner 2022). The metaphors the mind offers in this case are temporal.

## The progression of vulnerability framework: A rapid literature review

**FIGURE 1:**
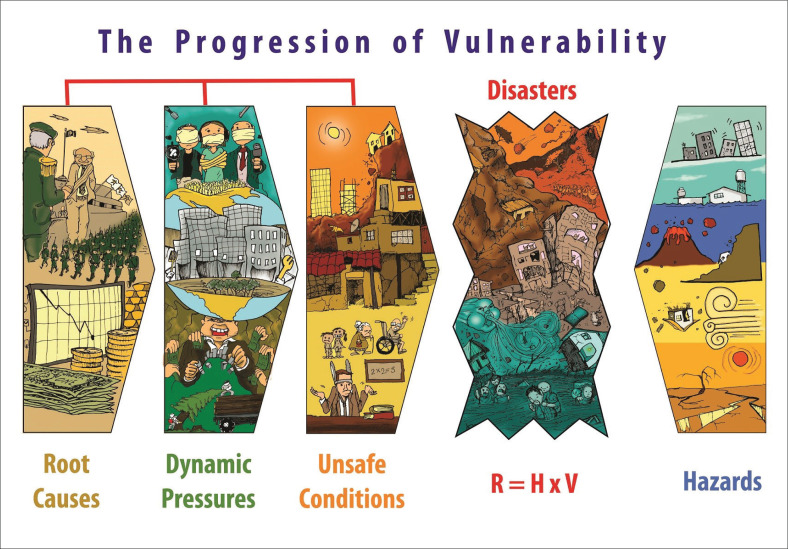
The ‘progression of vulnerability’ (progression of vulnerability) framework (illustration and design by Alejandro Katsumi Lemus and Irasema Alcántara-Ayala).

The objective of this rapid review of literature is to map the evolution of the impact of the PAR framework in the disaster risk reduction-related research and practice that has been reported in academic publications. In particular, this review focuses on identifying the core topics on which the PAR model has been used or theoretically considered as an insight for the development of different works (Figure 1).

The records used were obtained from the Scopus database, which is more comprehensive than the well-known Web of Science and has no language restrictions. The search was undertaken within the Scopus section ‘References’ that includes the name of the publication in which the PAR first was released (Blaikie et al. 1994). The first and subsequent editions of the book were considered in the analysis.

As of August 2022, the literature search resulted in 5472 research papers in the Scopus database. Results were exported to EndNote software where 49 duplicate articles were identified and removed. A total of 5423 publications met the inclusion criteria. The software WordStat 9 (Provalis Research 2021) was used to conduct a quantitative content analysis of the titles and abstracts of the selected papers.

Although the highest percentage of publications that refer to the PAR framework is in English, it has also been referenced in various other languages including Spanish and Chinese.

**FIGURE 2:**
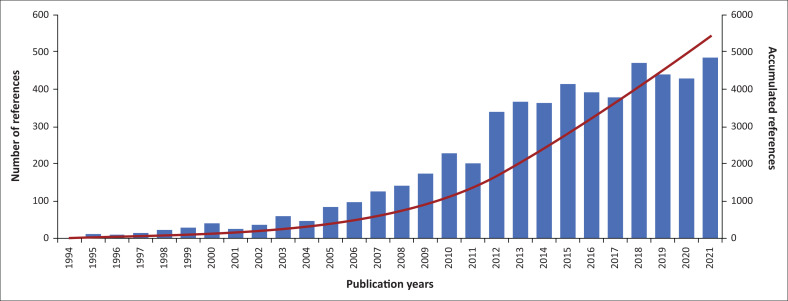
Temporal distribution of the references made to the progression of vulnerability framework during 1994–2021.

As shown in Figure 2, the temporal distribution of the references made to the PAR framework has increased over time. There was a clear boost from 2011 to 2012 when the number of references increased by 170%.

The first identified reference was published in the same year the PAR framework was released. It focused on the analysis of the concept of vulnerability, which was applied to a case study of refugee vulnerability in Greece. The study recognised that in addition to the characteristics of individuals and households, consideration must be given to the context of state policies and the wider characteristics of society and the economy to understand the dimensions of vulnerability (Black 1994).

During the following year, in 1995, a series of papers considered the PAR framework. Chan (1995) investigated the effectiveness of government-run permanent relocation schemes in response to flood hazards in Malaysia, while Chiotti and Johnston (1995) were already concerned about global environmental change in the context of farm adaptation. They suggested that a better understanding of the nexus between climate change and farm adaptation requires an approach that situates farm-level decision-making within structural forces, including biophysical ones.

Also in 1995, eight out of 13 papers comprised in the special volume of the *GeoJournal* entitled ‘Questioning Development: Growth? – Destruction? – Sustainability?’ (Vol. 35, No. 2, February 1995), included insights from the PAR framework (Ford & Adamson 1995; Handmer 1995; Parker & Mitchell 1995; Parker & Tapsell 1995; Ribot 1995; Wisner 1995; Wisner & Luce 1995; Yapa et al. 1995).

**FIGURE 3:**
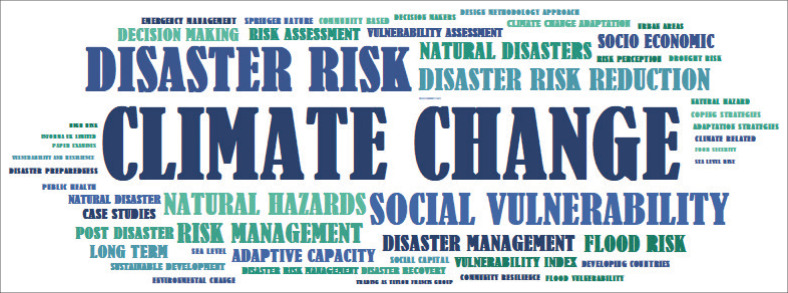
Visualisation of the most frequent words used in abstracts of the publications referring to the progression of vulnerability framework.

Over the years, the core research themes for which the PAR framework has been used or referenced have evolved along with concerns, concepts, perspectives, terminology and even buzzwords. Climate change, disaster risk, social vulnerability, disaster risk reduction, natural hazards and risk management were at the top of the most frequent keywords included in the abstracts of the reviewed publications. They were followed by terms such as natural disasters, flood risk, disaster management, socio-economic, adaptive capacity, long term, decision making, post-disaster, risk assessment vulnerability index, vulnerability assessment, sustainable development and climate change adaptation (Figure 3).

**FIGURE 4:**
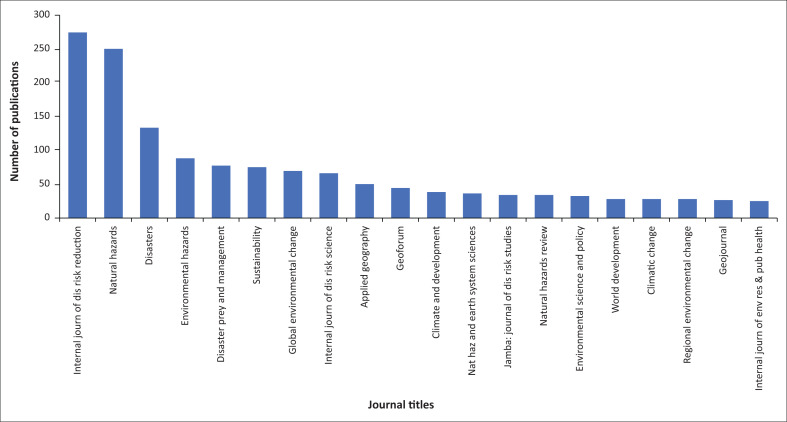
List of journals where the largest amount of research was published contemplating the progression of vulnerability framework.

The journals in which the largest amount of PAR-relevant research was published were the *International Journal of Disaster Risk Reduction, Natural Hazards* and *Disasters* (Figure 4). All of them are indexed in the Web of Science database, which only includes peer-reviewed publications.

**FIGURE 5:**
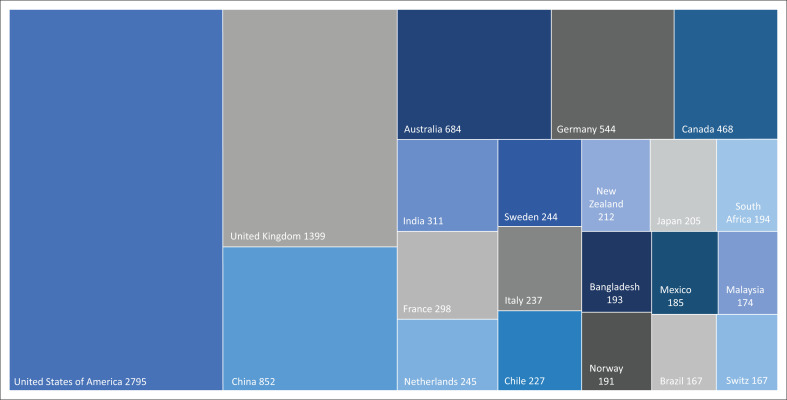
Frequency of publications referring to the progression of vulnerability framework by country of affiliation of the author(s).

The examination of the publications by geographical location shows the concentration of a higher frequency of references to the PAR framework in 21 countries, in each of which at least 150 articles were published (Figure 5). Although this illustration does not in any way reflect the countries where the PAR framework is most recognised and valued, these figures provide an indicator of the geographical distribution of the research carried out and published by the main producers of articles and show that these include a few large regional scientific hubs in the United States of America, the United Kingdom, China and Australia.

**FIGURE 6:**
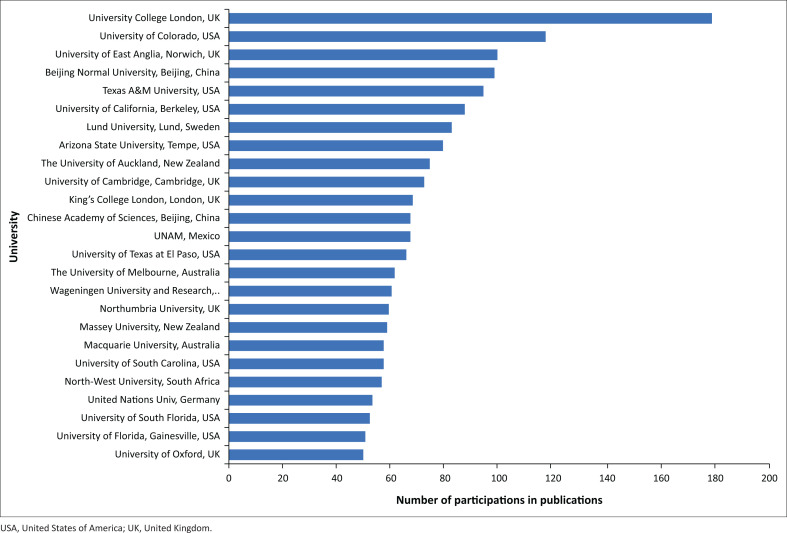
Number of participations in publications referring to the progression of vulnerability framework by institutions of the author(s).

Likewise, it was possible to identify a handful of academic institutions where the staff produced the largest number of publications referring to in the PAR framework. These were University College London (UK), University of Colorado (USA), University of East Anglia (UK), Beijing Normal University (China) and Texas A&M University (USA). Other universities and research institutions with high rates of publication are also included in Figure 6.

The results from this rapid literature review showed the frequency of themes addressed by the research in which reference is made to the PAR framework. Key issues involved the following:

1. Disaster risk reduction (53.11%).
2. Social vulnerability index (41.95%).
3. Social and economic dimensions (37.14%).
4. Disaster recovery (28.97%).
5. Climate change (27.75%).
6. Extreme weather events (27.27%).
7. Local and national government.
8. Adaptive capacity.
9. Theoretical issues of disaster research (13.53%).
10. Food security and water resources (13.46%).
11. Urban areas and settlements (11.36%).
12. Decision making (10.49%).

A systematic review of the literature using the PAR framework is beyond the scope of the present effort. For example, supplementary searches of other bibliographic databases would provide documents included in the so-called grey literature. A search on Google Scholar for the same period gives approximately 13 500 documents that cite the PAR framework. Much of this is grey literature would most likely contain clues to whether and how PAR has been used by governments, non-governmental organisations and international organisations.

## The other contributions

Irasema Alcántara-Ayala and her colleagues review several applications of their 2016 guidelines for the forensic analysis of disasters (FORIN) (Oliver-Smith et al. 2016). The use of the term ‘forensic’ refers to a systematic and analytical investigation to seek root causes and drivers of disasters and disaster risk. They argue that within the context of skewed, class-biased development practices that continue to affect all societies there is a need to address the meandering interplay that shapes the social construction of risk. The FORIN approach also enables researchers and practitioners to explore alternative opportunities for addressing interactions and interdependencies among disaster risk drivers and for challenging complexity. The FORIN perspective has much to offer as an overall strategy to stimulate the policymaking domain to understand causes and effects. Much work is still being done. For instance, Arabella Fraser headed a team that applied FORIN to a number of flood-prone situations.

The Preparing for Extreme And Rare events in coastaL regions project (PEARL) framework developed by Fraser provides an additional view of root causes (Fraser et al. 2020). It broadens the perspective covered by FORIN regarding governance processes and states that disaster ‘events’ are not discrete and unique; disasters concatenate and cascade, influencing existing risk accumulation processes. PEARL makes clear that the structure of vulnerabilities and disaster risk experiences interconnect with politics, and therefore, with the meanings and constructs of the spatial and temporal spheres of policymaking. Interconnection extends to multi-scale and multi-sectoral stakeholders and the influence of informal institutional practices. Thus, PEARL justifies working from disaster risk management ‘out’ and also from development practices ‘in’.

Both reflections on FORIN and PEARL cite an earlier framework as influential. This is the PAR published by Blaikie et al. (1994) and revised by Wisner et al. (2004). Progression of vulnerability is colloquially known as the ‘crunch’ framework because root causes are shown to be transmitted and modified by dynamic pressures that produce unsafe conditions when they encounter stress from a hazard. In its common representation, root causes and the rest of the cascade come from the left-hand side and hazards from the right. In his deeply critical assessment, Malm (in this volume) finds that PAR does not accommodate the challenge of climate change and needs adjustment ‘on the right’.

Loic Le De and colleagues return to their paper ‘Alternatives for sustained disaster reduction’ and ask why it is that its French version, although more detailed and extensive than the English, has failed to provoke much response among Francophone authors.

Taking a different approach to reflection, two groups of authors report bibliometric studies of the kind that spurred the curiosity of Le De’s group. B.L. Turner and Li & Ali Jamshed et al. take a systematic and quantitative approach to assess how the frameworks with which they are associated have been received and utilised over the years. Their results are consistent with those of Le De’s group. In all three cases, the reception of a social interpretation of risk and hazard has been uneven. In France, none but a young generation of researchers explore the way that socio-economic and demographic differences influence exposure to risk, the consequences of disasters on lives and livelihoods and on recovery outcomes.

The model developed by Turner et al. (2003a, 2003b) under the auspices of the US National Academy of Sciences highlights components of social and environmental subsystems and their interactions. However, as it has been applied, the focus has privileged social versus socio-environmental vulnerability. Many of the studies that cite Turner et al. do not pay attention to the full array of dimensions included in the original framework (Turner and Li, this volume).

Jamshed et al. explore the applications and impact of a framework that was built to assist the development of integrated methods of assessment of vulnerability to natural and socio-natural hazards. This facilitating framework is called Methods for the Improvement of Vulnerability Assessment in Europe (MOVE) (Birkmann et al. 2013). In addition to assessing factors and dimensions of vulnerability, MOVE encourages context-specific studies of vulnerability and risk assessment. The long-term impact of MOVE on vulnerability and risk assessment was amplified by its integration into the framework used by the Intergovernmental Panel on Climate Change (IPCC) (Roy et al. 2021; Sharma & Ravindranath 2019).

Finally, Ian Christoplos and John Mitchell revisit their 2001 framing of disaster. Their reflection involves thinking again about their original call to ‘reframe’ disaster; thus, like a fun house mirror, they offer us the potential for a long, possibly indefinite, regression. At the end of that series, in their words, perhaps there are ‘changes in jargon and scope – content, not so much’. Are they saying that understanding disaster is a perennial challenge that each generation must face?

## Does theory save lives?

Returning to the question posed in our subtitle, one has to consider the application of frameworks to actions by state and non-state actors. Theory itself does not do anything, much less safe lives. Decisions, allocations of resources and actions save lives or are intended to. The question, therefore, is whether theories of disaster risk reduction affect the decisions, resource allocations and actions of state and non-state actors. From this point of view, the answer is yes. Certainly, the vocabulary is used by state and non-state actors (Kelman 2018). ‘Vulnerability’ in particular has been operationalised by such actors in the form of guidelines and standard operating procedures and protocols (Wisner 2016). One might inquire further whether these guidelines are followed, and generally, this seems to be so. However, limitations on resources may require prioritisation and triage among the potential beneficiaries of assistance with disaster risk preparedness, response and recovery (Mena 2020).

## Closing remarks

Disaster risk does not stand still. Disaster risk frameworks must apprehend this moving reality. The notions contained in such frameworks are meant to bring knowledge and action together. Action to reduce risk grows increasing complexity because of the many challenges such as conflict, migration and climate change facing governments. The frameworks reviewed in this volume of Jamba provide a touchstone to guide wise action based on the understanding root causes and drivers of disasters.

The pursuit of power in today’s global world and the inescapable political character of disaster risk hamper human progress. Notwithstanding that the official discursive door has been opened to advance the UN 2030 international efforts, including the sustainable development goals, the climate change agreement, the urban agenda, and the disaster risk reduction framework, the propositions put forth, however, stand challenged and neglected by governments. The insulation of economic interests and unethical values in bubbles of power continues to inhibit human rights and justice.

Disaster risk research has evolved and so has the interaction among relevant DRR stakeholders. Yet, few substantial shifts have been made to reduce and avoid the construction of new risks. The constant untying and reassembling of disaster risk frameworks have forged new insights. Further formulations on the way disaster risk are understood reveal complex interlinkages and interdependencies that cannot be addressed by simplistic narratives and gradual and disconnected actions. If something of this mindset can be allowed to permeate the policy-making spheres, then responsibility, accountability and effective disaster risk governance can bring voice and hope where a world of desires now still reigns.

## References

[CIT0001] Birkmann, J., Cardona, O.D., Carreño, M.L., Barbat, A.H., Pelling, M., Schneiderbauer, S. et al., 2013, ‘Framing vulnerability, risk and societal responses: The MOVE framework’, *Natural Hazards* 67(2), 193–211. 10.1007/s11069-013-0558-5

[CIT0002] Black, R., 1994, ‘Livelihoods under stress: A case study of refugee vulnerability in Greece’, *Journal of Refugee Studies* 7(4), 360–377. 10.1093/jrs/7.4.360

[CIT0003] Blaikie, P., Cannon, T., Davis, I. & Wisner, B., 1994, *At risk*, Routledge, London.

[CIT0004] Chan, N.W., 1995, ‘Flood disaster management in Malaysia: An evaluation of the effectiveness of government resettlement schemes’, *Disaster Prevention and Management: An International Journal* 4(4), 22–29. 10.1108/09653569510093405

[CIT0005] Chiotti, Q.P. & Johnston, T., 1995, ‘Extending the boundaries of climate change research: A discussion on agriculture’, *Journal of Rural Studies* 11(3), 335–350. 10.1016/0743-0167(95)00023-G

[CIT0006] Davis, I. & Alexander, D., 2022, *A glass half-full or half-empty: Dialogue on disaster risk reduction*. Institute for Risk and Disaster Risk Reduction, University College London, viewed 10 December 2022, from https://www.preventionweb.net/publication/glass-half-full-or-half-empty-dialogue-disaster-risk-reduction.

[CIT0007] Ford, R.E. & Adamson, K.T., 1995, ‘The population-environment nexus and vulnerability assessment in Africa’, *GeoJournal* 35(2), 207–216. 10.1007/BF00814067

[CIT0008] Fraser, A., Pelling, M., Scolobig, A. & Mavrogenis, S., 2020, ‘Relating root causes to local risk conditions: A comparative study of the institutional pathways to small-scale disasters in three urban flood contexts’, *Global Environmental Change* 63, 102102. 10.1016/j.gloenvcha.2020.102102

[CIT0009] Handmer, J.W., 1995, ‘Managing vulnerability in Sydney: Planning or providence?’ *GeoJournal* 37(3), 355–368.

[CIT0010] Kelman, I., 2013, ‘Negotiating disasters: Politics, representation, meanings’, *Disaster Prevention and Management* 22(4), 378–380.

[CIT0011] Kelman, I., 2018, ‘Lost for words amongst disaster risk science vocabulary?’, *International Journal of Disaster Risk Science* 9(3), 281–291. 10.1007/s13753-018-0188-3

[CIT0012] Mena, R., 2020, ‘Disasters in conflict: Understanding disaster governance, response, and risk reduction during high-intensity conflict in Afghanistan, South Sudan and Yemen’, PhD thesis, Erasmus University Rotterdam/International Institute of Social Studies.

[CIT0013] Oliver-Smith, A., Alcántara-Ayala, I., Burton, I. & Lavell, A., 2016, *Forensic investigations of disasters (FORIN): A conceptual framework and guide to research*, Integrated Research on Disaster Risk, Beijing.

[CIT0014] Parker, D. & Mitchell, J.K., 1995, ‘Disaster vulnerability of megacities: An expanding problem that requires rethinking and innovative responses’, *GeoJournal* 37, 295–301. 10.1007/BF00814008

[CIT0015] Parker, D. & Tapsell, S., 1995, ‘Hazard transformation and hazard management issues in the London megacity’, *GeoJournal* 37(3), 313–328. 10.1007/BF00814011

[CIT0016] Provalis Research, 2021, *WordStat 9, user’s guide [Text Analytics Software]*, viewed 10 December 2022, from https://provalisresearch.com/Documents/WordStat9.pdf.

[CIT0017] Revet, S. & Langumier, J., 2015, ‘Introduction’, in S. Revet & J. Langumier (eds.), *Governing disasters: Beyond risk culture*, pp. 1–20, Palgrave, London.

[CIT0018] Ribot, J.C., 1995, ‘The causal structure of vulnerability: Its application to climate impact analysis’, *GeoJournal* 35, 119–122. 10.1007/BF00814058

[CIT0019] Roy, B., Khan, M., Mostafa, S., Saiful Islam, A.K.M., Khan, M., Uddin, J. et al., 2021, ‘Integrated flood risk assessment of the Arial Khan River under changing climate using IPCC AR5 risk framework’, *Journal of Water and Climate Change* 12(7), 3421–3447. 10.2166/wcc.2021.341

[CIT0020] Sharma, J. & Ravindranath, N.H., 2019, ‘Applying IPCC 2014 framework for hazard-specific vulnerability assessment under climate change’, *Environmental Research Communications* 1(5), 051004. 10.1088/2515-7620/ab24ed

[CIT0021] Tronto, J., 1993, *Moral boundaries: A political argument for an ethic of care*, Routledge, London.

[CIT0022] Turner, B.L., Kasperson, R.E., Matson, P.A., McCarthy, J.J., Corell, R.W., Christensen, L. et al., 2003a, ‘A framework for vulnerability analysis in sustainability science’, *Proceedings of the National Academy of Sciences* 100(14), 8074–8079. 10.1073/pnas.1231335100 PMC166184 12792023

[CIT0023] Turner, B.L., Matson, P.A., McCarthy, J.J., Corell, R.W., Christensen, L., Eckley, N. et al., 2003b, ‘Illustrating the coupled human–environment system for vulnerability analysis: Three case studies’, *Proceedings of the National Academy of Sciences* 100(14), 8080. 10.1073/pnas.1231334100 PMC166185 12815106

[CIT0024] Wisner, B., 1988, *Power and need in Africa*, Earthscan, London.

[CIT0025] Wisner, B., 1995, ‘Bridging “expert” and “local” knowledge for counter-disaster planning in urban South Africa’, *GeoJournal* 37(3), 335–348. 10.1007/BF00814014

[CIT0026] Wisner, B., 2016, *Vulnerability as concept, metric, model and tool*, Oxford University Press, viewed 8 December 2022, from https://oxfordre.com/naturalhazardscience/view/10.1093/acrefore/9780199389407.001.0001/acrefore-9780199389407-e-25?mediaType=Article.

[CIT0027] Wisner, B., 2022, ‘Power writ small and large: How disaster cannot be understood without reference to pushing, pulling, coercing, and seducing’, in G. Bankoff & D. Hilhorst (eds.), *Why vulnerability still matters: The politics of disaster risk creation*, pp. 171–191, Routledge, London.

[CIT0028] Wisner, B., Blaikie, P., Cannon, T. & Davis, I., 2014, *At risk: Natural hazards, people’s vulnerability and disasters*, 2nd edn., Routledge, London.

[CIT0029] Wisner, B., 1995, ‘Questioning development – Growth? Destruction? Sustainability?’, *GeoJournal* 35, 99–104. 10.1007/BF00814056

[CIT0030] World Health Organization and Pan American Health Organization, 2022, *Emergencies/supply management*, viewed 13 February 2023, from https://www.paho.org/en/topics/emergencies.

[CIT0031] Yapa, L., Wisner, B. & Luce, H.R., 1995, ‘Building a case against economic development’, *GeoJournal* 35(2), 105–118. 10.1007/BF00814057

